# Development of a speckle-based portable device for *in situ* metrology of synchrotron X-ray mirrors

**DOI:** 10.1107/S1600577516012509

**Published:** 2016-08-16

**Authors:** Yogesh Kashyap, Hongchang Wang, Kawal Sawhney

**Affiliations:** a Diamond Light Source, Harwell Science and Innovation Campus, Didcot, Oxfordshire OX11 0DE, UK

**Keywords:** speckle, X-ray optics, metrology

## Abstract

A portable device for *in situ* metrology of synchrotron X-ray mirrors based on the near-field speckle scanning technique has been developed. Ultra-high angular sensitivity is achieved by scanning a piece of abrasive paper or filter membrane in the X-ray beam. In addition to the compact setup and ease of implementation, a user-friendly graphical user interface has been developed to ensure that optimizing active X-ray mirrors is simple and fast. The functionality and feasibility of this device have been demonstrated by characterizing and optimizing X-ray mirrors.

## Abstract 2

A portable device, using the speckle scanning technique, has been developed for *in situ* metrology of X-ray mirrors at synchrotron light facilities.

## 1. Introduction   

The successful exploitation of X-ray beams generated by modern third-generation synchrotron light sources, such as Diamond Light Source (Diamond), depends to a significant extent on developments in X-ray optics. Due to their achromaticity and large acceptance aperture, X-ray mirrors are widely used at synchrotron light facilities for micro- and nano-focusing. In order to generate either focused or defocused beams, X-ray active mirrors, such as bimorph and mechanically bendable mirrors, are used on almost all beamlines at Diamond. Although *ex situ* measurement of X-ray mirrors using interferometry and deflectometry are routinely performed (Qian *et al.*, 1995 ▸; Siewert *et al.*, 2004 ▸; Alcock *et al.*, 2010 ▸, 2015 ▸), the ultimate performance of X-ray mirrors is critically dependent on the exact nature of the working conditions, such as ultra-high vacuum, photon-induced heat load, mechanical clamping and vibrations (Wang *et al.*, 2013 ▸; Rutishauser *et al.*, 2013 ▸). Therefore, it is equally important to perform *in situ* characterization and optimization of X-ray mirrors under beamline conditions. Not all beamlines are equipped with sufficient diagnostics, which motivates the creation of a portable *in situ* metrology device for use on a range of beamlines. Accurate *in situ* metrology is also essential to achieve diffraction-limited and coherence-preserved beams (Sawhney *et al.*, 2013 ▸). This situation will become increasingly important as synchrotron light sources are upgraded to further improve the photon beam brilliance. Over the last two decades, several *in situ* metrology techniques have been developed to evaluate the performance of various X-ray optics (Sutter *et al.*, 2012 ▸; Hignette *et al.*, 1997 ▸; Wang *et al.*, 2011 ▸, 2014 ▸; Idir *et al.*, 2010 ▸). The speckle scanning technique shows great potential for practical application since it requires a simple experimental setup and has less stringent requirements for spatial coherence of the X-ray beam. Importantly, ultra-high angular sensitivity has been demonstrated using the speckle scanning technique.

In order to perform *in situ* characterization of mirrors at different hard X-ray beamlines, in this study we present the development and implementation of a portable metrology device based on the X-ray speckle scanning approach. A graphical user interface (GUI) has been designed to enable easy and quick data analysis. We demonstrate the performance of this device by investigating and optimizing X-ray mirrors.

## 2. Principle   

The device is based on the principle of X-ray near-field speckle, which has previously been used in X-ray phase contrast imaging, coherence measurements and mirror metrology (Berujon *et al.*, 2014 ▸; Alaimo *et al.*, 2009 ▸; Wang *et al.*, 2016 ▸; Wang, Kashyap *et al.*, 2015 ▸; Wang, Berujon *et al.*, 2015 ▸; Kashyap *et al.*, 2015 ▸). When a monochromatic, partially coherent X-ray beam is passed through a random medium, a speckle pattern is observed in the near-field. Each speckle pattern is unique, and this remarkable property allows its use as a wavefront marker. The basic principle of speckle scanning is to measure the deflection of near-field speckles in the presence of optical elements. Although it is essential to place the diffuser (abrasive paper or filter membrane) upstream of the test mirror to obtain a quantitative slope error map, it is usually difficult to implement this scheme due to space constrains and sealed vacuum vessels of pre-installed X-ray mirrors. Moreover, for most practical cases, wavefront errors and the focal size of the X-ray beam are of most interest to the beamlines. Therefore, we have developed a portable device operating downstream of the test mirror which characterizes the total wavefront distortion caused by all upstream components. When the diffuser is placed downstream of the mirror, the second derivative of the wavefront, namely the inverse of its local radius of curvature (wavefront slope error), is usually measured. This takes account of imperfections introduced to the wavefront not only by the mirror but also by any optics upstream from the point of measurement. In this configuration, we mount a sheet of abrasive paper downstream of the mirror under test, and the wavefront slope error is measured by scanning the abrasive paper transversely through the X-ray beam. In addition, a pair of crossed-wires can also be mounted beside the diffuser to precisely measure the X-ray focal spot.

**Figure 1:**
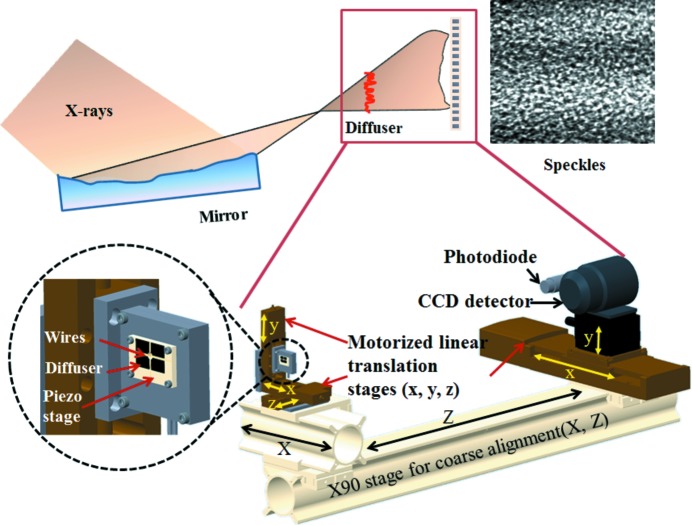
Schematic mechanical and optical layout of the *in situ* portable device for characterization of X-ray optics. The diffuser, wires, CCD detector and photodiode can be translated into the X-ray beam using motorized linear stages. The wavefront is measured by scanning the diffuser with a piezo stage, whereas the beam size at the focal position can be measured *via* a knife-edge scan using the wires and photodiode.

The optical layout of the experimental setup is illustrated in Fig. 1 ▸. As the diffuser is scanned perpendicular to the beam direction, speckle patterns recorded by the detector undergo displacement which depends upon the wavefront distortion introduced by the mirror. Let 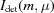 be the detector signal recorded as a function of the piezo scanning step (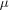) at the detector pixel row *m*. The delay signal 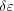 can be recovered by locating the peak of maximum correlation in the cross-correlation of pixels *m* and 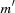, respectively, using the following relation (Pan *et al.*, 2006 ▸),
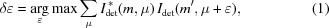
where 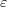 is the signal delay parameter. The maximum signal delay obtained by cross-correlation can be directly related to the inverse of the radius of curvature 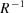 through the following relation (Berujon *et al.*, 2012 ▸),
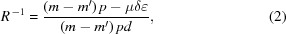
where *d* is the distance between the diffuser and detector, and *p* is the detector pixel size. Once the inverse of radius of curvature 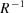 is derived, the wavefront slope φ along the horizontal or vertical directions can be calculated. It may also be noted that the radius of curvature is a scalar field whereas the slope is a vector field. Therefore, it is more straightforward to use the wavefront radius of curvature rather than the wavefront slope as a merit parameter for mirror optimization. The wavefront radius of curvature will be perfectly spherical for the ideal point focus case, and the measured error of the wavefront radius of curvature 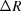 will be related to imperfections on the measured mirrors and upstream beam aberrations. Moreover, for the optimization of bimorph mirrors, the piezo response functions (PRF) in terms of the local curvature can be approximated by Gaussian functions, whereas those measured in terms of slope resemble non-analytical error functions. Therefore, it is more accurate and efficient to use the local inverse radius of curvature of the wavefront to generate PRF instead of using the wavefront slope (Wang *et al.*, 2015 ▸).

## 3. Design of the *in situ* metrology device   

### 3.1. Hardware design   

**Figure 2:**
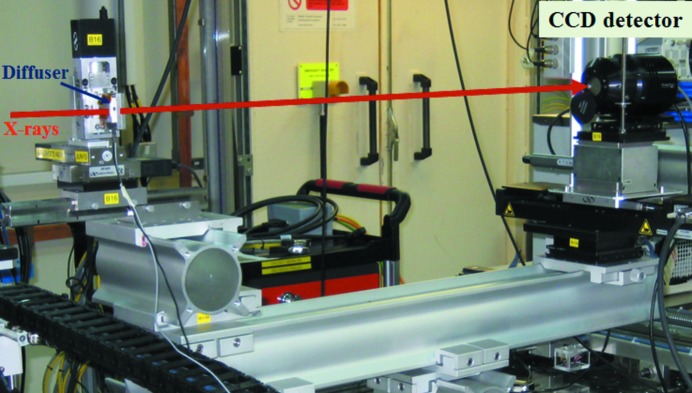
Photograph of the portable metrology device installed on B16 beamline for the characterization of X-ray mirrors. The mirror is installed upstream of the membrane stage and is therefore not visible in the above figure.

A schematic of the mechanical layout of the device is also shown in Fig. 1 ▸. The entire setup has been purposefully designed on a modular base frame (Newport X90 stage) for coarse alignment and ease of portability. Such a frame can readily be fitted onto virtually any beamline. The diffuser is mounted on a piezo stage for precision scanning, which in turn is mounted on an assembly of three linear stages (25 mm travel range) for alignment of the diffuser with the direct or reference X-ray beam. In addition, a pair of crossed gold wires with a diameter of 200 µm is also attached to the piezo stage to permit measurement of the X-ray beam-size. Coarse alignment is performed manually, and the distance between the mirror focus and the diffuser can be freely chosen so as to optimize the angular sensitivity. Further downstream, a CCD detector with pixel size of 6.5 µm is used to record the speckle pattern. A photodiode detector was also mounted on the detector stage to perform knife-edge scan and measure X-ray beam size. Both detectors are mounted on horizontal and vertical motorized translation stages for ease of alignment with the X-ray beam. All motorized stages are remotely controlled with an accuracy of 1 µm *via* the Experimental Physics and Industrial Control System (EPICS) based on a Geo Brick LV system (Dalesio *et al.*, 1994 ▸). The device is capable of measuring either horizontal or vertical reflection mirrors by scanning the piezo stage in the appropriate direction. This feature is especially useful if there is a need to characterize composite optics, such as Kirkpatrick–Baez mirrors. X-ray experiments were conducted at the Test beamline B16 at Diamond (Sawhney *et al.*, 2013 ▸) to test the functionality of the device. Fig. 2 ▸ shows a photograph of the experimental assembly as installed on the beamline.

### 3.2. Design of data acquisition and GUI   

**Figure 3:**
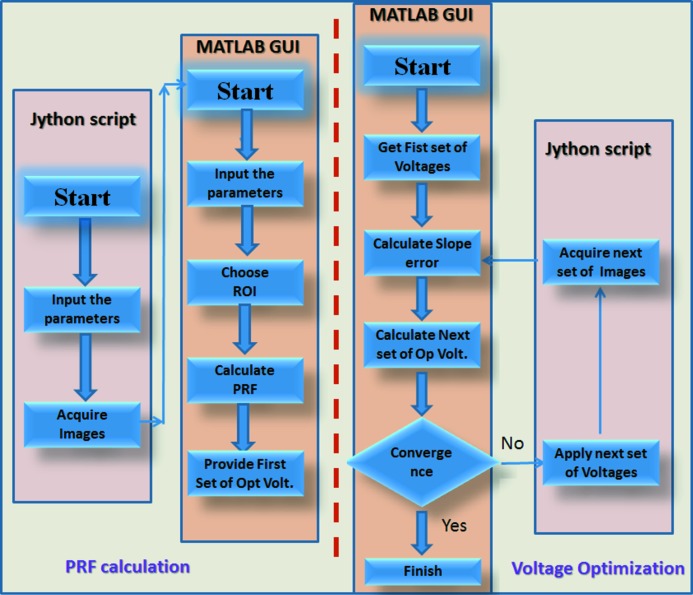
Flow chart of the implementation of the partially automated optimization procedure for piezo bimorph mirrors using the speckle scanning approach.

**Figure 4:**
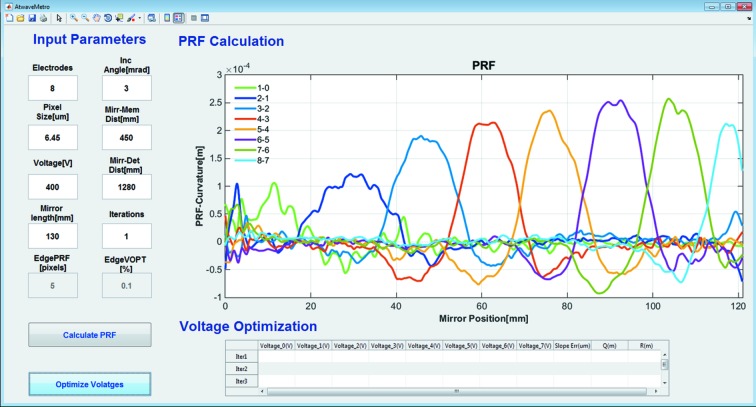
MATLAB GUI for calculating the piezo response functions (PRF) and voltage optimization of a bimorph mirror. The snapshot shows the localized wavefront curvature change induced by applying a fixed voltage to each individual piezo electrode. Here 1-0 represents the differential response of electrodes as measured before and after applying a voltage.

Data acquisition and analysis software is currently split into two parts. Fig. 3 ▸ shows a flow chart of the software used for mirror optimization. Motorized stages at the beamline are controlled through a distributed, heterogeneous computing environment, using embedded systems for direct control of instrumentation, together with Linux workstations for the user interfaces. These are connected across a network to workstations that provide the user interface and other functionality. The software development for the control system is built on the EPICS control system tool kit which is an open-source software platform used by many large-scale facilities. A Jython script is used to control data acquisition *via* the Generic Data Acquisition (GDA), which is an open-source framework used at all Diamond’s beamlines. Based on user inputs of acquisition time, piezo-step size, piezo-voltage increment, number of scan points *etc.*, the Jython script collects a series of images at different voltage settings of the bimorph mirror’s piezo electrodes. A standalone MATLAB GUI is used to calculate how each of the bimorph’s piezos responds to an applied voltage, the so-called piezo-response functions (PRF), by subtracting the values of wavefront slope (or inverse of radius of curvature) extracted from the *j*th to (*j* − 1)th measurement. The typical time for wavefront reconstruction is a few minutes. Fig. 4 ▸ shows a snapshot of the MATLAB GUI used for calculating the PRF and voltage optimization. After deriving the PRF, the first set of optimized voltages is automatically calculated and displayed on the GUI for user convenience. Values are also archived for further processing. To reduce the mirror’s slope error, voltages generated in the first iteration are applied to relevant electrodes, and another stack of speckle images is collected to evaluate the new error of the wavefront radius of curvature for a second iteration. This process is repeated until convergence occurs. In practise, two or three iterations are typically sufficient to minimize the slope error and obtain the optimum set of voltages that give the best X-ray focus.

## 4. Experimental results   

To investigate the functionality of the device, an elliptical mirror was characterized using the developed portable device by placing the diffuser downstream of the test mirror, and the results were also compared with the *ex situ* measurement from the Diamond-NOM slope profilometer (Alcock *et al.*, 2010 ▸). The design parameters of the mirror are: source-to-mirror distance *p* = 41.5 m, mirror-to-focus distance *q* = 0.1 m, and grazing incidence angle θ = 3 mrad. The polished region (70 mm) of the mirror was fully illuminated with monochromatic 15 keV X-rays from a double-crystal monochromator (DCM). 80 images with a step size of 0.25 µm were collected to measure the tangential wavefront slope error for both *in situ* measurements. It should be noted that the wavefront error is determined on a propagation plane perpendicular to the optical axis of the reflected beam at the mirror centre. In order to convert the measured wavefront error into a mirror slope error, the wavefront error profile has to be projected to the mirror surface by taking into account the mirror slope and the angle variation of the reflected beam (Shi *et al.*, 2014 ▸).

**Figure 5:**
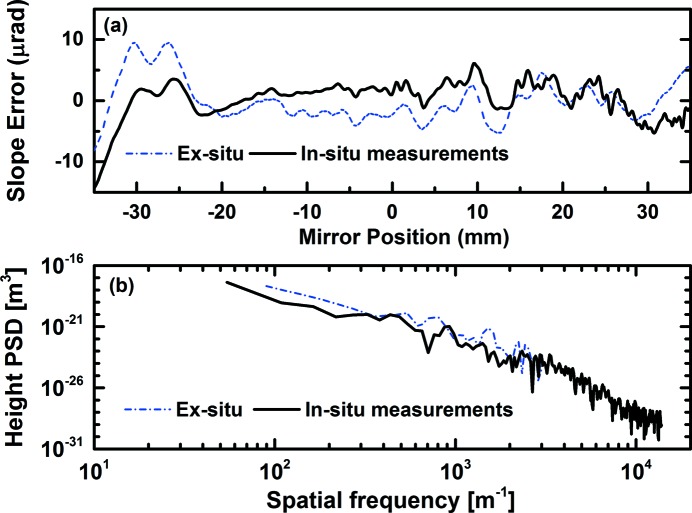
Comparison of the (*a*) slope error and (*b*) height power spectral density of an elliptical test mirror as measured using: *in situ* portable device (black, solid); and *ex situ* profilometry using the Diamond-NOM (blue, dash-dot).

As seen in Fig. 5(*a*) ▸, the *in situ* metrology measurements with the portable device are compared with the *ex situ* Diamond-NOM data. Although the major features of optical polishing errors are in good agreement between the *ex situ* and *in situ* metrology techniques, some discrepancy in the low-frequency region can still be noticed. One possible reason is that an ellipse was removed from slope data from the Diamond-NOM, whereas only a simple linear fit is used to derive the wavefront slope error. Another reason is that the difference between the wavefront slope error and mirror slope error cannot be assumed simply to be equal to each other and an accurate wave-optical calculation is needed to map the wavefront slope errors into the mirror shape error (Yumoto *et al.*, 2006 ▸). Here, we would like to emphasize that the effective spatial resolution for *in situ* metrology is about 0.2 mm thanks to the large geometrical magnification, while the spatial resolution of the Diamond-NOM is only a few millimetres. A comparison of Diamond-NOM measurement and speckle measurements in terms of power spectrum density is shown in Fig. 5(*b*) ▸, and indicates that the portable device can provide the slope error information (or height error) in the middle-frequency regions, which is often difficult to access for conventional visible-light metrology techniques.

**Figure 6:**
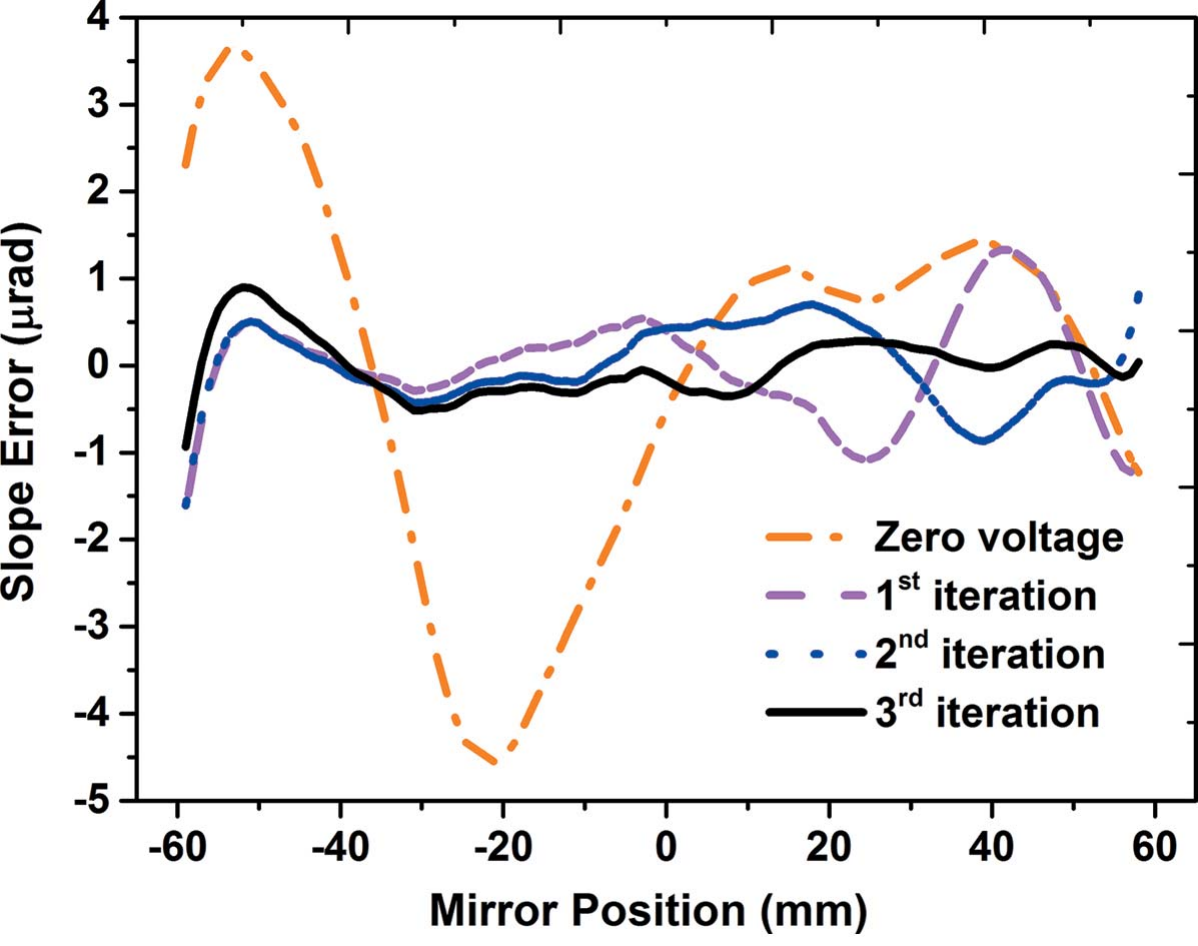
The optical slope error of the bimorph mirror was reduced from 2.3 µrad at zero voltage (orange, dash-dot) to 0.2 µrad (solid, black) in three iterations.

To assess the feasibility of using the portable device for optimizing an active X-ray mirror, a deformable piezo bimorph mirror was investigated. The eight-electrode bimorph, manufactured by Thales-SESO (France) and super-polished by elastic emission machining (EEM) over an active length of 120 mm at JTEC (Japan), has an elliptical shape with: *p* = 41.5 m, *q* = 0.4 m and θ = 3 mrad. The mirror was mounted on a motorized tower in the experimental hutch of B16 at 47 m from the X-ray source. Since the mirror substrate is uncoated silica, X-rays with energy of 9.2 keV were selected by the DCM. PRF were obtained by incrementally applying 400 V to each piezo electrode. As shown in Fig. 2 ▸, the portable system, consisting of the diffuser and detector, was mounted downstream of the test mirror on an optical table. For each set of applied voltages, 80 images were collected by scanning the membrane in steps of 0.25 µm and the data collection time for each image was 1 s. The membrane was scanned vertically (tangential slope error) only because sagittal slope errors have much less effect due to the grazing incidence of the X-ray beam. As discussed, the PRF were determined in terms of inverse of radius of curvature for fast optimization and convergence. Fig. 6 ▸ shows the measured wavefront slope error after application of voltages obtained for successive iterations. Slope error was reduced from 2.3 µrad (r.m.s) to 0.2 µrad (r.m.s) in three iterations.

**Figure 7:**
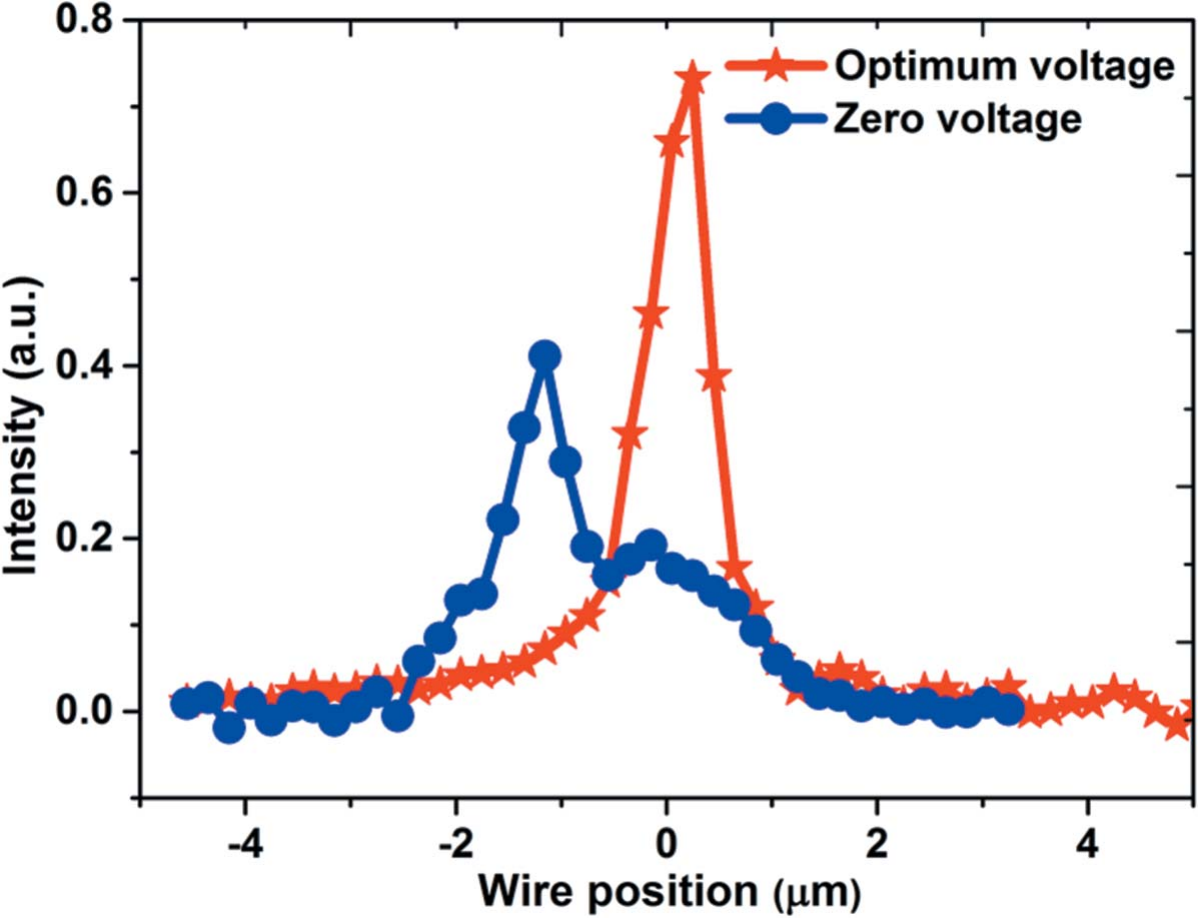
First derivative of the transmission signal from a gold wire scan in the focal plane of the bimorph mirror for zero voltage (blue, circles) and optimized voltages (red, stars) showing the improvement to the size and shape of the X-ray beam.

It should be emphasized that the sensitivity of the wavefront slope error measurement using near-field speckle is dependent on various factors including tracking accuracy, step size and distance between the membrane and detector. For the presented instrument, the maximum detector-to-mirror distance is 1275 mm, and the tracking accuracy is 0.05 pixel according to the visibility of the recorded speckle pattern. Hence, an angular sensitivity of ∼10 nrad can be achieved with a step size of 0.25 µm (Wang, Sutter *et al.*, 2015 ▸). Angular sensitivity can be further enhanced by improving the speckle quality and increasing the mirror-to-detector distance, or utilizing a better speckle tracking algorithm. Therefore, such a portable device offers very high sensitivity for *in situ* mirror optimization. To verify the optimization results, the beam size was also measured by performing a knife-edge scan by translating the gold wires in 0.1 µm steps through the focal plane and measuring the X-ray intensity with a diode detector. Fig. 7 ▸ shows the first derivative of the transmission signal from the wire scan in the focal plane of the bimorph mirror for zero and optimized voltages. The success of optimizing the bimorph mirror using feedback from the portable device was clearly demonstrated by reducing the X-ray focal spot size from 2.5 µm to 0.6 µm.

## 5. Summary   

We have developed a portable metrology device for *in situ* characterization and optimization of X-ray mirrors at Diamond Light Source. The user-friendly interface requires minimal user intervention during the optimization process. We demonstrate that the best focus can be achieved within a few iterations for a bimorph mirror using this device and accompanying optimization software. This compact device can be easily implemented on a variety of operational beamlines, and provides wavefront error measurement and optimization of X-ray mirrors. Although two independent software modules are used to acquire and process data, we intend to streamline the process into a single software package for better compatibility and ease of operation. This fast, compact and accurate speckle-based device is expected to find wide application for *in situ* characterization of X-ray mirrors for many beamlines, both at Diamond Light Source and synchrotron light facilities elsewhere. It may be noted that such a portable device can also be used for measurement of beam coherence or characterization of other types of X-ray optics, such as compound refraction lens and Fresnel zone plates.
